# Transmission trends of the global COVID-19 pandemic with combined effects of adaptive behaviours and vaccination

**DOI:** 10.1017/S0950268823000274

**Published:** 2023-02-20

**Authors:** Yuhao Zhou, Zhaowan Li, Wei Wu, Jianpeng Xiao, Wenjun Ma, Guanghu Zhu

**Affiliations:** 1 School of Mathematics and Computing Science, Guangxi Colleges and Universities Key Laboratory of Data Analysis and Computation, Guilin University of Electronic Technology, Guilin, China; 2 Guangdong Provincial Institute of Public Health, Guangdong Provincial Center for Disease Control and Prevention, Guangzhou, China; 3 Department of Public Health and Preventive Medicine, School of Medicine, Jinan University, Guangzhou, China; 4 Center for Applied Mathematics of Guangxi (GUET), Guilin, China

**Keywords:** Adaptive behaviour, COVID-19, prediction, transmission model, vaccination

## Abstract

We developed a mechanism model which allows for simulating the novel coronavirus (COVID-19) transmission dynamics with the combined effects of human adaptive behaviours and vaccination, aiming at predicting the end time of COVID-19 infection in global scale. Based on the surveillance information (reported cases and vaccination data) between 22 January 2020 and 18 July 2022, we validated the model by Markov Chain Monte Carlo (MCMC) fitting method. We found that (1) if without adaptive behaviours, the epidemic could sweep the world in 2022 and 2023, causing 3.098 billion of human infections, which is 5.39 times of current number; (2) 645 million people could be avoided from infection due to vaccination; and (3) in current scenarios of protective behaviours and vaccination, infection cases would increase slowly, levelling off around 2023, and it would end completely in June 2025, causing 1.024 billion infections, with 12.5 million death. Our findings suggest that vaccination and the collective protection behaviour remain the key determinants against the global process of COVID-19 transmission.

## Introduction

The novel coronavirus (COVID-19) has been raging around the world for 3 years and still stops gaining momentum. The cumulative number of global cases exceeded 661 million as of December 2022, which is posing big burdens on population health and economic development [1]. Various intervention measures were taken to different degrees around the world, but due to the variability and high pathogenicity of the virus and limited protection of vaccines, disease control continues to be an arduous mission. Everyone is eagerly awaiting for its ending day. In this paper, we assess the intervention outcomes and estimate the trends of global infection under different scenarios of collective behaviours.

The ongoing outbreak of COVID-19 has triggered a range of protective behaviours in the human population (such as social distancing, wearing mask, keeping hygienic and vaccination) [2]. Designing effective intervention strategies (e.g. consideration of psychosocial factors [3], behaviour change [4] and vaccination patterns [5, 6]) has gained significant attention. Recent studies found that wearing masks, maintaining social distance and good hygiene practices play roles in reducing the infection risk. Many studies have been conducted regarding vaccination evaluation, such as prioritising vaccination for different age groups [7]; predicting the epidemic end time in the USA [8]; estimating the number of infections, hospitalisations and deaths avoided by vaccine [9]; and predicting the end time of Omicron variant [10]. Some epidemiology experts believe that COVID-19 could coexist with humans for a long time, unless a highly effective vaccine emerges [11].

Dynamical models based on compartmental principle have been widely applied to tackle COVID-19 transmission problem [12, 13]. Using mathematical equations to simulate population transfer among different states from macroscopic perspective, it enables to reveal the mechanistic patterns of disease transmission. For example, by building a SEIR model with intervention effects, it was found that self-awareness and government-imposed measures can effectively mitigate and delay infection [4]; by integrating intervention measures into a SEIR model and fitting the surveillance data in China and Korea, it was found that continuous and integrated interventions (especially population screening) are the epidemic control key [14]; by combining asymptomatic infection into a SEIAR model with vaccination and family structure, it was found that vaccination alone could not control the epidemic [15]; and by fitting the incidence data through a SVEIH model, it was estimated that vaccines enabled 1.5 million infections to be avoided [16]. Here the capitals in the model denote the population that are susceptible (S), exposed (E), infectious with symptom (I), infectious and either asymptomatic or with very mild symptoms (A) and infectious that require hospitalisation (H). In this paper, we went a further step to consider the following question: how to use dynamic models to portray the mutual coupling mechanisms of disease, population adaptive behaviour and vaccination, and thus predict the end time of COVID-19 infection?

To address the above question, we first developed an ordinary differential system with SVEIR compartments to simulate COVID-19 transmission, in which vaccination reduces the probability of being infected and adaptive behaviours modulate infectivity. We then explored the propagation dynamic features. We finally applied the model to analyse the global data by using MCMC method to fit uncertain model parameters. By doing so, we evaluated the effects of vaccination and adaptive behaviours, and predicted the end time of this epidemic under different conditions in global scale.

## Method

### Model framework

This is a computational study. Based on the existing knowledge of the clinical progression of COVID-19 infection, we developed a new transmission model by using ordinary differential equations and then applied this model to simulate disease evolution. Humans are classified into different categories according to their infection status: susceptible (*S*), latent (*E*), asymptomatic infection (*I*_*b*_), clinical infection (*I*_*c*_), vaccinated (*V*), recovered (*R*) and dead (*D*). The sum of these classes (except *D*) equals the total population size, i.e. *N* = *S* + *E* + *I*_*b*_ + *I*_*c*_ + *Ic* + *R* + *V*.

**Fig. 1.**
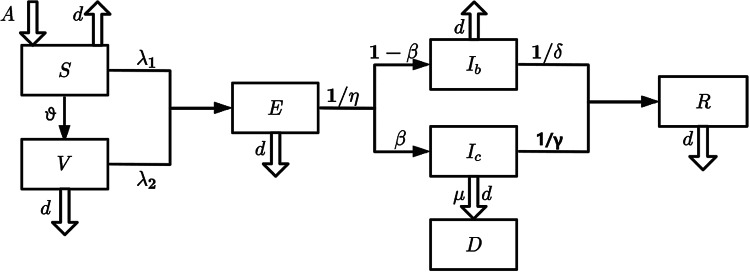
Flow diagram of COVID-19 transmission.

Newly infected individuals are generated on account of exposure to infectious individuals. A susceptible population (*S*) can be infected at rate *λ*_1_ through contact with infectious individuals (*I*_*b*_ and *I*_*c*_). It then transforms into a latent state (*E*) and becomes infectious after an incubation period 1/*η*. Parts of them (accounting for *β* proportion) become clinically infected, and the others become asymptomatic. People can acquire immunity through vaccination and then move from susceptible state to vaccinated state. Known that vaccine only reduces the risk of infection, those vaccinated population (*V*) will be infected at a lower rate *λ*_2_ after exposure to infectious population. Asymptomatic infected individuals (*I*_*b*_) are not easily detected and treated, but they can recover after period 1/*δ*. Clinically infected individuals (*I*_*c*_) recover after an average treatment period 1/*γ*. The corresponding flow chart is shown in Figure 1, which is formulated by a differential system in the model (1).1
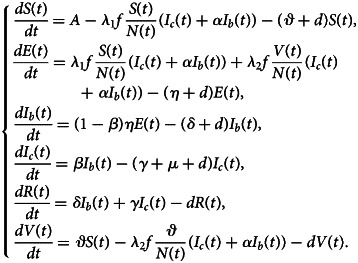

The combined effect of vaccine 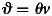 is the product of the vaccination rate *v* and the vaccine potency *θ*. Here our model equalised the effectiveness of different vaccines in the world and regarded them as a whole.

The adaptive behaviours are considered in the following ways. During the COVID-19 pandemic, people usually change their behaviour and take preventative steps to reduce infection risk, such as social distancing and wearing masks. Individuals' self-protection awareness would intensify as the infection cases increase, and then they would adopt stricter measures against infection. We called such performances as adaptive behaviours. They are a collective designation of protection actions which are subject to people's self-protection awareness. Given that adaptive behaviours can reduce infection risk, they are quantified as a coefficient of infectivity, which is a decreasing function of case number, written as *f*(*I*,  *R*). According to the expression given in reference [17], we set *f* = (1 − (*I* + *ϕR*)/*N*)^*k*^. Parameter *k* measures the magnitude of adaptive behaviour, in which *k* = 0 indicates no adaptive behaviour, and a larger *k* indicates that people are more sensitive and cautious to the infection situation. Parameter *ϕ* ∈ [0,  1] represents the sensitivity of humans to the number of historical cases, where a larger *ϕ* indicates a stronger long-term awareness of protection in the population.

### Parameter setting and data analysis

The total population of the world is set to be *N* = 7.898 billion. Birth constant *A* was estimated by using model *dN*/*dt* = *A* − *dN* to fit the world population during 2012 and 2021. The vaccination rate *v* is determined by the proportion of the average vaccinated population. The vaccine efficacy is first set to be *θ* = 60% for validating the model, and then we changed its value and run the model for observing the outcomes of different vaccine efficacies. The numbers of vaccinated people and reported cases per day were extracted from the website (see Supplementary Material) [18]. The time span is from 22 January 2020 to 18 July 2022, with a total of *T*  =  909 days. Considering the existence of changes in population behaviour and virus variation during epidemic evolution, infection rates (*λ*_1_,  *λ*_2_) and adaptive parameters (*ϕ*,  *k*) vary during three periods with different main strains. Model parameters are shown in Tables 1 and 2.

**Table 1.** Parameter introduction

Parameters	Definitions	Values	Source
1/*η*	Incubation time of the virus	3	[19]
1/*δ*	Time from onset to treatment	14	[20]
*θ*	Vaccine effectiveness rate	60%	[21]
1/*γ*	Time to treatment for clinically infected patients	19	[22]
1/*d*	Average life expectancy (days)	28 215	[23]
*μ*	Mortality rate of clinically infected patients per unit of time	0.011–0.035	[24]
*v*	Rate of vaccination	0–0.096%	[18]
*A*	Birth constant	8.408 × 10^7^	Estimation

**Table 2.** Parameter changes before and after vaccination

	Pre-vaccination		Post-vaccination		Basic reproduction number *R*_0_
Time span	Infection rate *λ*_1_	Adaptive protection *k*	Infection rate *λ*_2_	Adaptive protection *k*	
Initial strain	0.14	1.2	0.07	1.1	3.28
Delta	0.18	45	0.09	35	4.23
Omicron	0.2	85	0.1	80	5.01

We applied the proposed model to fit the global COVID-19 infection data from 22 January 2020 to 18 July 2022. To take into account the different infectivity from different viral strains, we divided the time period into three segments according to the changes of main virus strains: initial strain, Delta and Omicron, and then we estimated their respective infectivity. MCMC algorithm was used to estimate unknown parameters (including *k*, *λ*_1_,  *λ*_2_,  *ϕ* and the initial values of *E*), which was achieved by using the FME package in R. Considering the protective effect of vaccine, we assumed that vaccination halves the infection rate, that is *λ*_2_ = 0.5*λ*_1_. The initial values of model variables were set equal to the reported number in initial time. We examined the model performance quantified by minimum fit error criterion. The error is determined by 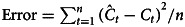, where *C*_*t*_ (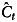) is the numbers of reported cases (modelling cases) on day *t*.

## Results

**Fig. 2.**
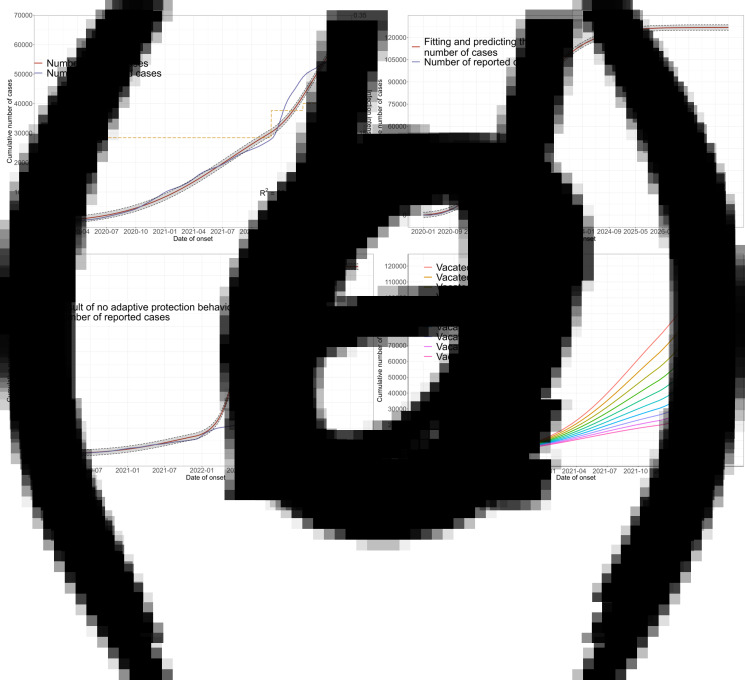
Simulations of COVID-19 transmission under different scenarios by using the model: (a) fitting results; (b) predicting transmission trend; (c) transmission without adaptive protective behaviour; (d) transmission with different vaccine protection rates.

We used the theory of next-generation matrix to calculate the basic reproduction number *R*_0_, one of the most important theoretical concepts in epidemiology to quantify the infection potential [25], that is,
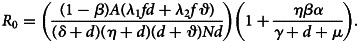


The fitting result is shown in Figure 2(a), where the model results and the number of reported cases overlap well, with *R*^2^ = 93%. Yet, the fitting curve is a little higher than the reported cases since May 2022. The principal reason could be that since many countries eased intervention policies, parts of cases were undetected and unreported. During the prevalence periods of the initial strain, Delta and Omicron, the estimated infection rate *λ*_1_ increased from 0.14 to 0.18 and to 0.20; long-term awareness parameter *ϕ* decayed from 0.5 to 0.02 and to 0.01; the basic reproduction number is 3.28, 4.23 and 5.01, respectively. The morality rate *μ* during the phases of initial, Delta and Omicron strains was estimated to be 2.8%, 3.5% and 1.14%, respectively.

The simulation results of without protective behaviours are shown in Figure 2(c). It is observed that this case would result in 1.992 billion infections by 18 July 2022, and the epidemic could stop by October 2023. By then the number of infections would reach 3.098 billion, which is 5.39 times of the current infection number.

The simulations of epidemic transmission with different vaccine protection rates are shown in Figure 2(d). It is observed that without vaccination, the cumulative number of cases would reach 1.215 billion by 18 July 2022, which means that vaccination prevents 645 million people from being infected. If the vaccine protection rate is reduced to 10% or increased to 90%, the cumulative number of cases would reach 1.095 billion or 369 million, which is 192% or 64% of the current number of infections.

**Fig. 3.**
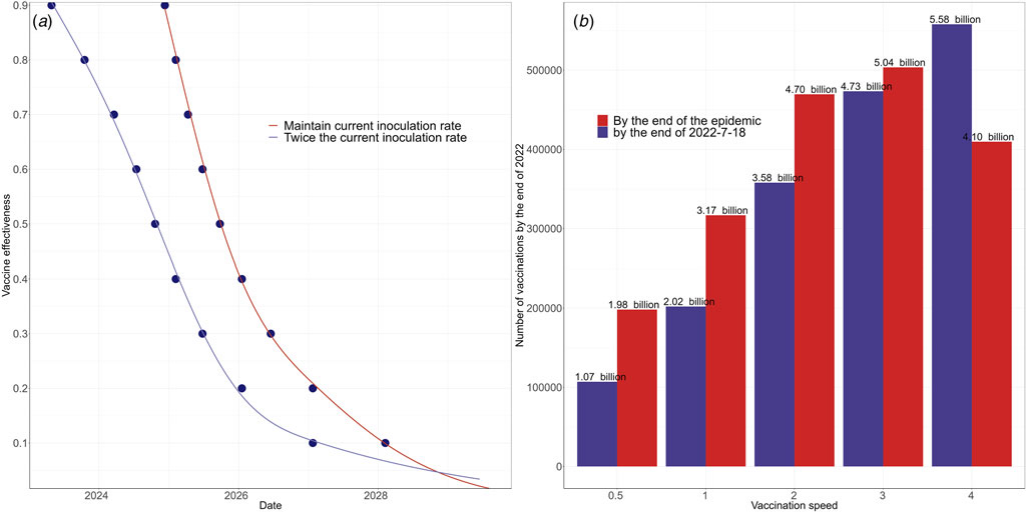
Vaccine requirements for stopping COVID-19 transmission, estimated by using the proposed model: (a) the relationship between epidemic end and vaccine effectiveness; (b) number of vaccines required for different vaccination rates.

The simulation results of predicting the epidemic transmission trend are shown in Figures 2(b) and 3(a), and Table 3. It is found that if the global vaccination rate is maintained at current level (3 787 200 vaccinations per day on average) and the vaccine protection rate and adaptive parameters remain unchanged, then the number of infections would slow down and level off by the end of 2023. In this case, the epidemic would completely end in June 2025, leading to 1.024 billion of cumulative cases, with 12.5 million death.

**Table 3.** Effect of vaccine efficiency

	Maintaining current inoculation rate			Twice current inoculation rate		
Efficiency (%)	Cumulative cases (10^9^)	End time	Duration (days)	Cumulative cases (10^9^)	End time	Duration (days)
0	21.34	2031/10/11	4281	21.34	2031/10/11	4281
10	18.57	2028/02/07	2939	16.48	2027/01/24	2560
20	16.48	2027/01/24	2560	13.07	2026/01/18	2189
30	14.68	2026/06/18	2340	10.24	2025/06/26	1983
40	13.07	2026/01/18	2189	7.79	2025/02/06	1843
50	11.60	2025/09/25	2074	5.65	2024/10/21	1735
60	10.24	2025/06/26	1983	3.79	2024/07/15	1637
70	8.97	2025/04/11	1907	2.31	2024/03/20	1520
80	7.79	2025/02/06	1843	1.39	2023/10/18	1366
90	6.69	2024/12/11	1786	0.94	2023/04/28	1193

Table 3 illustrates the transmission outcomes under different vaccine patterns. If the vaccine protection rate increases from 0.6 to 0.7, 0.8 and 0.9, the end of the epidemic would advance to April 2025, February 2025 and December 2024, causing 897, 779 and 669 million infections, respectively. If the vaccination rate is doubled, the epidemic would completely end in October 2024, causing 379 million infections. In this case, increasing the vaccine efficacy to 0.7, 0.8 and 0.9 would advance the end to March 2024, October 2023 and April 2023, causing 231, 139 and 94 million infections, respectively. In particular, epidemic duration is highly correlated with vaccine protection rate, with correlation coefficients as −0.84. The results of fitting their relationship by using a generalised linear model are shown in Figure 3(a), where the epidemic duration increases approximately linearly as the vaccine protection rate decreases.

Global production of COVID-19 vaccine doses reached 11 billion doses by the end of 2021, with 9 billion doses expected to be produced in 2022, so the supply of vaccines is adequate. Disregarding the global wealth gap and specific vaccine supply measures, we use the prior vaccination rate as the baseline value to examine the changes in the epidemic at different vaccination rates. As shown in Figure 3(b), the vaccine doses required until the end of the epidemic would not exceed the number of vaccines currently available [26].

Under current conditions of vaccine efficacy and adaptive behaviour, the impacts of vaccination rates are shown in Table 4 and Figure 3(b). It is observed that fast vaccination can result in short transmission duration. If halving vaccination rate, the end of the epidemic would be delayed until the end of June 2026, causing 1.468 billion cases. If the vaccination rate is increased to 2–4 times, the end of the epidemic would advance to between July 2024 and October 2021, causing 49–279 million cases. As shown in Figure 3(b), increasing the rate of vaccination could not cause the lack of vaccine supply.

**Table 4.** Effect of vaccination rate

Speed	Cumulative infection (10^9^ doses)	End time	Duration (days)	Vaccine demand (10^9^ doses)
0.5	14.68	2026/06/18	2340	1.98
1	10.24	2025/06/26	1983	3.17
2	3.79	2024/07/15	1637	4.70
3	0.93	2023/04/28	1193	5.04
4	0.49	2021/10/24	642	5.58

From the sensitivity analysis (Supplementary Fig. S1), it is observed that the most sensitive parameters to model output are infection rate *λ*_1_, adaptive parameter *k*_1_ and vaccination rate *v*. The partial rank correlation coefficient (PRCC) values for parameters *λ*_1_,   *λ*_2_,   *k*_1_,   *k*_2_,   *v* and *θ* were 0.98, 0.18, −0.42, –0.08, −0.88 and −0.18, respectively. While the PRCC value corresponding to *ϕ* is not significant.

## Discussion

We have developed a deterministic model by using a system of differential equations to simulate COVID-19 transmission. The model incorporates adaptive behaviour of human population and the protective effect of vaccine. Based on the daily vaccination data and the cumulative number of reported cases, we applied MCMC method to estimate model parameters, from which we evaluated the effects of adaptive behaviours and vaccination, and further predicted the end time of the COVID-19 pandemic around the world.

First, the protective behaviour of populations during epidemic periods of different strains was assessed. Using the model to simulate the long-term transmission process, it was found that the absence of adaptive behaviours could result in 25.2% of the global population being infected by 18 July 2022. In this case, herd immunity could be reached around October 2023, but the number of infected people would be 3.098 billion by then, which is about 39.26% of the total global population. Many studies have confirmed that non-pharmaceutical interventions played important roles in epidemic control, possibly leading to a weakening transmission by about 20–70% [4]. This paper went a further step to evaluate the intervention outcome from a global scale. The results also indicated the key role of non-pharmaceutical interventions.

Second, vaccination can effectively control the spread of COVID-19. Our results showed that as of 18 July 2022, if without vaccination, the number of global infections could be 1.215 billion, that is, vaccination could avoid 52% infections. This is consistent with a recent study, in which the authors claimed that vaccine avoids 52% infections in the USA as of September 2021 [9]. Several other studies have focused on the protective effect of vaccines against fatal cases [9]. Most studies emphasised that vaccination should be intensified and vaccine effect should be optimised in time. Doing so can effectively avoid the re-emergence of outbreaks, and also bring forward the epidemic end.

Finally, adaptive behaviours and vaccine interventions can modify COVID-19 transmission trends. Simulation results indicated that in the absence of adaptive behaviours, the pandemic could end in October 2023, with 39% of global population being infected. To eradicate the epidemic, without reducing the intensity of adaptive behaviour in humans, it should ensure a large vaccination rate with a high level of vaccine efficacy. If the vaccination rate is expanded by 1–3 times, the end time would advance to July 2024, April 2023 and October 2021. The increase in the vaccination rate does not result in an oversupply of vaccine. If the vaccine protection rate is increased to 0.7, 0.8 and 0.9, the end time would advance to March 2024, October 2023 and April 2024. Regarding this, the UK government gave projections for optimistic, intermediate and pessimistic scenarios in November 2021, ending in 2022–23, 2023–24 and 2026, respectively. A quite recent study used a SEIR model to estimate the pandemic ends in several countries and in the world, predicting it around November 2023 [10]. Increasing vaccination rates, optimising vaccine efficacy and increasing protection awareness can effectively reduce human infections and disease duration. These measures should be pursued simultaneously in this anti-epidemic battle.

This paper has the following limitations: (1) the results are based on the simple assumptions of transmission model, which may yield deviation from reality due to the complexity in the real world; (2) the numbers of cases and vaccinations are based on publicly available data, which may differ from reality; (3) the model does not take into account reinfection and future viral strain variation, as well as the impacts of vaccines on mortality; and (4) since we define the end time of infection as the day when no new cases are generated by running the proposed model, which may yield deviation due to numerical calculation error and large scale of modelling data. Yet, the model takes into account the variability of viral strains and the impact of vaccines on epidemics, as well as the dynamics of adaptive behaviour in humans. Historical incidence data were used as a reference to fit the model with an accuracy of 93%, which laid the foundation for the subsequent analysis and convinced that the model is consistent with current situation.

## Data Availability

All data are available in Supplementary Material.

## Supplementary material

For supplementary material accompanying this paper visit https://doi.org/10.1017/S0950268823000274. click here to view supplementary material
