# Supplementary material for: Transmission trends of the global COVID-19 pandemic with combined effects of adaptive behaviours and vaccination
Source: Epidemiol Infect. 2023 Feb 20;151:e39. doi: 10.1017/S0950268823000274 (PMC10024953; doi:10.1017/S0950268823000274)
Supplement: Supplementary file 1 [file hygsup.zip › S0950268823000274sup002.docx]

**Figure S1.** Sensitivity of model parameters by PRCC values.
